# Cell-Crossing Functional Network Driven by microRNA-125a Regulates Endothelial Permeability and Monocyte Trafficking in Acute Inflammation

**DOI:** 10.3389/fimmu.2022.826047

**Published:** 2022-03-24

**Authors:** Martin Bernhard Müller, Max Hübner, Lei Li, Stephanie Tomasi, Valena Ließke, David Effinger, Simon Hirschberger, Kristin Pogoda, Markus Sperandio, Simone Kreth

**Affiliations:** ^1^ Walter Brendel Center of Experimental Medicine (WBex), Ludwig Maximilians University München (LMU), Munich, Germany; ^2^ Department of Anaesthesiology and Intensive Care Medicine, Research Unit Molecular Medicine, LMU University Hospital, Ludwig Maximilians University München (LMU), Munich, Germany; ^3^ Department of Transfusion Medicine, Cell Therapeutics and Haemostaseology, LMU University Hospital, Ludwig Maximilians University München Ludwig Maximilians University (LMU): Munich, Munich, Germany; ^4^ Physiology, Institute for Theoretical Medicine, University of Augsburg, Augsburg, Germany; ^5^ Biomedical Center (BMC), Institute for Cardiovascular Physiology and Pathophysiology, Walter Brendel Center for Experimental Medicine (WBex), Ludwig Maximilians University München, Faculty of Medicine, Munich, Germany

**Keywords:** microRNA network, inflammation, immune cell trafficking, monocytes, endothelial cells

## Abstract

Opening of the endothelial barrier and targeted infiltration of leukocytes into the affected tissue are hallmarks of the inflammatory response. The molecular mechanisms regulating these processes are still widely elusive. In this study, we elucidate a novel regulatory network, in which miR-125a acts as a central hub that regulates and synchronizes both endothelial barrier permeability and monocyte migration. We found that inflammatory stimulation of endothelial cells induces miR-125a expression, which consecutively inhibits a regulatory network consisting of the two adhesion molecules VE-Cadherin (CDH5) and Claudin-5 (CLDN5), two regulatory tyrosine phosphatases (PTPN1, PPP1CA) and the transcription factor ETS1 eventually leading to the opening of the endothelial barrier. Moreover, under the influence of miR-125a, endothelial expression of the chemokine CCL2, the most predominant ligand for the monocytic chemokine receptor CCR2, was strongly enhanced. In monocytes, on the other hand, we detected markedly repressed expression levels of miR-125a upon inflammatory stimulation. This induced a forced expression of its direct target gene CCR2, entailing a strongly enhanced monocyte chemotaxis. Collectively, cell-type-specific differential expression of miR-125a forms a synergistic functional network controlling monocyte trafficking across the endothelial barrier towards the site of inflammation. In addition to the known mechanism of miRNAs being shuttled between cells *via* extracellular vesicles, our study uncovers a novel dimension of miRNA function: One miRNA, although disparately regulated in the cells involved, directs a biologic process in a synergistic and mutually reinforcing manner. These findings provide important new insights into the regulation of the inflammatory cascade and may be of great use for future clinical applications.

## Introduction

Infections, trauma and many other insults evoke expression and secretion of an elaborate sequence of inflammatory mediators that lead to the efficient recruitment of targeted immune cells to the affected site. Activation of the inflammatory cascade aims at limiting the spread of pathogens and tissue damage, initiating tissue repair, and eventually ending up with resolution. There are various plasmatic and cellular systems involved in orchestrating these fine-tuned processes including not only immune cells but also the endothelial cell layer, which increases its permeability thereby opening the barrier for leukocytes to infiltrate the tissue and resolve the menacing noxa. However, dysregulation of these inflammatory reactions can lead to life-threatening diseases with massive organ damage, as seen in sepsis and SIRS (1, 2). Hallmark of these conditions is a hyperpermeable endothelial barrier leading to circulatory break-down and massive accumulation of undirected dysfunctional immune cells in the tissue. How these processes are regulated still remains elusive, and deeper insights into the underlying molecular mechanisms are urgently necessary to develop new targeted therapy concepts.

The role of microRNAs (miRNAs) as regulators of inflammation has been well defined during the last decade. Innumerable single miRNA-target interactions have been identified to exert regulatory effects in specific cell types (3–5). In the last few years, however, it has become increasingly clear that the full regulatory impact of miRNAs is displayed by complex cell-crossing regulatory networks, where individual miRNAs target multiple genes in different cell types, thereby potentiating their impact on cellular functions (6). MiRNA networks that regulate the inflammatory cascade on a cell-type-spanning level have not been identified, yet. We explored the question of whether a specific miRNA could fulfil such a function as a master regulator of the inflammatory cascade across cell types.

We here uncover a miR-125a-driven functional network that impacts two different cell entities, endothelial cells and monocytes, resulting in synergistic effects within the inflammatory cascade. Upon inflammatory stimuli, miR-125a up-regulation in endothelial cells decreases the expression of five target molecules regulating the endothelial barrier thereby increasing its permeability. Simultaneously, miR-125a down-regulation in monocytes leads to forced expression of chemokine receptor 2 (CCR2), which induces monocyte trafficking towards the site of inflammation.

The here presented cell-spanning network is a new way of functional regulation, which fundamentally differs from the known concept of miRNAs being shuttled between cells *via* extracellular vesicles. Based on disparate expression patterns, miR-125a synergistically orchestrates the inflammatory cascade in endothelial cells and monocytes in response to inflammatory cytokines.

## Materials and Methods

### Cell Culture

Primary Human Umbilical Vein Endothelial Cells (HUVEC) were isolated from umbilical cords of healthy neonates directly after cesarean delivery at the Department of Gynecology and Obstetrics, University Hospital, LMU Munich, Germany. Written informed consent from the mother was obtained before donating umbilical cords in accordance with the Declaration of Helsinki. HUVEC were isolated from umbilical vein vascular wall by collagenase A (Roche) treatment and cultured in Endothelial Cell Basal Medium (ECGM; PromoCell) with SupplementMix (PromoCell), 10% FCS (Biochrom AG) and 1% Penicillin/Streptomycin (Pen/Strep, Gibco). Cells of one single cord at passage 2-4 were used for each of the independent experiments.

HEK-293 cells were purchased from the American Type Cell Culture Collection and cultured in Dulbecco’s Modified Eagle Medium (Gibco) supplied with 10% FCS, 2% L-Glutamine, (Gibco) 1% Penicillin/Streptomycin (Gibco) and 1% MEM NEAA (Gibco). HEK-293 cells used for experiments were not cultured beyond passage 20. Monocytes were maintained in RPMI (Lonza) supplemented with 20% FCS, 1% Penicillin/Streptomycin/Glutamine, 1% HEPES (Gibco). All cells were cultured at 37°C and 5% CO2.

### Isolation of Primary Human Monocytes, Inflammatory Stimulation and Differentiation

PBMCs were isolated from Lithium-heparinized freshly drawn blood of healthy volunteers with Histopaque 1077 (Sigma) according to manufacturer’s instructions. Thereafter, monocytes were extracted using the Pan Monocyte Isolation Kit II, human (Miltenyi) according to the instructions. For inflammatory stimulation, monocytes were immediately seeded in RPMI media supplemented with 50ng/ml IL-6 (Miltenyi) and compared to monocytes incubated without IL-6. IL-6 was used to mimic early inflammatory conditions as IL-6 clinically precedes acute phase proteins occurring in the blood of patients with acute inflammatory diseases (7). Monocytes show potent endothelial adhesion and transmigration upon IL-6 stimulation (8).

For macrophage differentiation, primary monocytes were incubated in RPMI supplemented with 50ng/ml M- or GM-CSF (Miltenyi) for 6 days. Media was changed every 2 days.

For investigation of miR-125a induction in HUVEC, cells were cultivated until monolayers reached 80% confluency and serum-starved with ECGM containing 0.5% FCS for 12 hours at 37°C. The culture medium was removed and cells were incubated in ECGM with 25ng/ml TNF and 50ng/ml IFN-γ (both Miltenyi). The combination of TNF and IFN-γ is known as a potent inducer of proinflammatory micro-RNAs (9, 10).

To address the early phase of inflammation, stimulation time-points were chosen between 3-4 hours.

### Transfections

Transfections were conducted using the NEON electroporation device (Life Technologies) according to the manufacturer’s protocol. When HUVEC reached 80-90% confluency, cells were detached and transfected with Ambion^®^ hsa-miR-125 premiR™, hsa-miR-125 mirVana™ miRNA inhibitor (miR-125 inhibitor; both Thermo Fisher) or respective siRNA (Dharmacon). Transfections were carried out at final concentrations of 50nM (premiR™) or 100nM (miR-125a inhibitor, siRNA). Electroporation for HUVEC was carried out using 1 pulse of 1350 Volt and 30 ms.

For monocytes, transient transfections of miRNA mimic (premiR™) and siRNAs were performed using the NEON electroporation device at a cell density of 2x10^6^ cells and a final concentration of 50nM premiR™ or 100nM siRNA per transfection. Transfection efficiency of miRNAs was determined by flow cytometry using Cy3-labeled premiR™ negative control (Thermo Fisher). Knockdown efficiency of siRNAs was evaluated by qRT-PCR or SDS-PAGE. Cell viability was assessed after electroporation with 50 nM premiR™ negative control using flow cytometry of propidium iodide stained cells. Viability of monocytes was between 70 to 83% and viability of HUVEC was between 81 to 89%.

### ECIS

For electric cell-substrate impedance sensing (ECIS) measurements, transfected HUVEC were seeded at a density of 100,000 cells/well into gelatin-coated electric cell-substrate impedance sensing arrays, each containing 8 wells with 40 gold electrodes per well (ECIS 8W10E+ PET; Ibidi). After 48 hours of incubation, cells were stimulated with TNF (25 ng/ml) for 24h. Impedance was measured directly after seeding for 72h with multiple frequencies mode using the ECIS^®^Z system (Applied BioPhysics). For analysis of barrier function, the area under the curve (AUC) of normalized impedance values over time at 4,000 Hz was calculated.

### FITC-BSA Passage

Transendothelial passage of macromolecules was assessed by measuring the passage of Fluorescein isothiocyanate conjugate bovine serum albumin (FITC-BSA, 66 kDa; Sigma-Aldrich) across confluent HUVEC monolayers. HUVEC were cultured in 24-well plates containing cell culture inserts (pore size 0.4 μm, Greiner Bio-one) with 200 μl ECGM growth medium in the upper chamber and 800 µl ECGM growth medium in the lower chamber. 48 hours after transfection, HUVEC were stimulated with 25ng/ml TNF for 24 hours. Subsequently, 10μg FITC-BSA was added to the upper compartment of each insert and the medium of bottom wells was collected after 30 minutes. Fluorescence intensity was measured using a FilterMax F3 (Molecular Devices) using Fluorescence Intensity (FL) read mode with an excitation wavelength of 485 nm and an emission wavelength of 535 nm.

### Quantitative Real-Time PCR

Total RNA was isolated from HUVEC or primary human monocytes using the miRNeasy Mini kit (Qiagen) according to the manufacturer’s protocol. RNA amount and quality was assessed using a NanoDrop 2000 spectrophotometer (Thermo Fisher) and reversely transcribed to cDNA using Oligo-dT Primers, Random Hexamers (Qiagen), dNTPs, RNAse OUT, and Superscript^®^ III Reverse Transcriptase (Invitrogen). Quantitative real-time PCR (qRT-PCR) was performed using specially designed primers (Probe Finder Software: Roche; primers synthesized by Metabion) and UPL Probes (Roche). All analyses were performed in duplicates on a Light Cycler 480 instrument (Roche) with 10ng of cDNA/well. For HUVEC, Glyceraldehyde 3-phosphate dehydrogenase (GAPDH) and TATA Box Binding Protein (TBP) were used as reference genes. For monocytes, Beta-2 Microglobulin (B2M) and TBP served as reference genes. QRT-PCR was conducted as previously described (11). Quantification cycle values were calculated by the “second derivative maximum” method computed by the LightCycler^®^ software. Primer sequences are supplied in **Supplementary Table S1**.

For quantifying miRNA expression, total RNA was reversely transcribed as previously described (12), using the specific primers of the TaqMan MicroRNA Assay for miR-125a-5p (Assay ID 002198, Applied Biosystems). Expression of miR-125a-5p was studied by qRT-PCR using the TaqMan MicroRNA Assay with U47 (Assay ID 001223, Applied Biosystems) as the reference RNA. Taqman qRT-PCR conditions comprised initial denaturation for 10 minutes (95°C), and 50 cycles of 95°C for 15 s, 60°C for 60 s; 40°C for 30 s.

### Next Generation Sequencing and Bioinformatics Analysis

For transcriptome analysis, primary HUVEC from five different donors were transfected in five independent experiments using either hsa-miR125-5p Pre-miR miRNA or negative control and cultivated for 18 hours. After additional stimulation with TNF (25ng/ml) RNA was isolated and analyzed by IMGM Laboratories GmbH (Martinsried). Quality control for all total RNA samples was done on the 2100 Bioanalyzer (Agilent Technologies) using RNA 6000 Nano LabChip Kits (Agilent Technologies). Library preparation was performed with the TruSeq^®^ Stranded mRNA HT technology (Illumina), according to the manufacturer’s protocol, including fragmentation, a poly-T oligo pulldown and sequencing adapter ligation. RNA sequencing was performed on the Illumina NextSeq^®^ 500 next generation sequencing system and its high output mode with 1 x 75 bp single-end read chemistry with >10 million reads per sample. For RNA Seq analysis and heatmap generation CLC Genomics Workbench and Bioconductor/R v3.0.2 was used. Differentially expressed genes were calculated by using non-corrected p-value < 0.01 and fold change +1.25 to -1.25 in order to identify potential targets which were further validated using qRT-PCR and western blots. RNA-Seq data is available on the Gene Expression Omnibus under the accession number: GSE196161 (https://www.ncbi.nlm.nih.gov/geo/query/acc.cgi?acc=GSE196161).

Analysis of potential miR-mRNA interactions was performed using the public databases TargetScan7.2 (http://www.targetscan.org), and miRIAD (http://bmi.ana.med.uni-muenchen.de/miriad/). Gene Set Enrichment Analysis was performed with the investigating gene sets function on http://www.gsea-msigdb.org/gsea/msigdb/annotate.jsp. Briefly, we computed overlaps between the list of differentially expressed genes found in Next Generation Sequencing with previously mentioned filtering strategy and the gene sets canonical pathways_KEGG and gene ontology_biological processes from the Molecular Signatures Database v7.5. Gene sets with a false discovery rate corrected p-values < 0.01 were seen as significant.

### SDS-PAGE

Cells were lysed in RIPA Lysis and Extraction Buffer (Thermo Fisher Scientific) containing 1% protease and phosphatase inhibitors (Cell Signaling Technologies). Protein concentrations were assessed through BCA assays (Thermo Fisher) according to the manufacturer’s instructions. Cell lysates were electrophoresed on SDS-PAGE gels and then electroblotted on polyvinylidene difluoride (PVDF) membranes using the Trans-Blot Turbo Transfer System (Biorad). Nonspecific binding was blocked with 5% non-fat milk or 5% Bovine Serum Albumin (BSA) in TBS-Tween-20 (TBST; Sigma) for 1 hour. Primary antibodies for VE-Cadherin (sc-9989), PTP1B (sc-133259), Claudin-5 (sc-374221), PP1α (sc-271762), Ets-1(sc-55581), TGFβ RII (sc-17792), Rac-2 (sc-517424) and α-actinin-4 (sc-393495) (all Santa Cruz Biotechnology) were diluted in TBST with 5% non-fat milk, Phospho-VE-Cadherin antibody (Tyr658, Cat. No.44-1144G; Thermo Fisher) was diluted in TBST with 5% BSA. β-Actin (#4970; Cell Signaling Technologies) served as the loading control. Immunoreactive bands were visualized by using peroxidase-conjugated secondary antibodies (Anti-rabbit IgG, #7074; Anti-mouse IgG, #7076; Cell Signaling Technologies) and the ECL western blot detection system (BioRad).

### Cloning of Reporter Constructs

The 3’UTRs of CDH5, PTPN1 and CCR2 were amplified from genomic DNA (50 ng) by PCR using the following cycling conditions and the primers listed in **Supplementary Table S2**. PCR products were ligated into the StrataClone Blunt Vector Arms (Agilent Technologies) according to manufacturer’s instructions and then subcloned into the psiCHECKTM2 vector (Promega) using XhoI/NotI restriction enzymes (New England Biolabs) and T4 DNA Ligase (Roche). Site-directed mutagenesis of plasmid DNA was conducted using the QuikChange Lightning Multi Site-Directed Mutagenesis Kit (Agilent Technologies) according to the manufacturer’s protocol. Sequences were verified by Sanger sequencing (Eurofins). Plasmids were purified using the Qiaprep Spin Plasmid Miniprep Kit (Qiagen) and the Pure Yield Plasmid Midiprep System (Promega). DNA concentrations were measured using a NanoDrop 2000 spectrophotometer (Thermo Fisher).

### Reporter Gene Assays

Co-transfection of luciferase reporter plasmids and premiR™ was carried out using 100,000 HEK-293 cells, 50 nM hsa-miR125 premiR™ and 1 µg of Psi-CHECK™2 plasmid, followed by 40 hours of incubation. Dual-Glo Luciferase Assay system (Promega) was used according to the manufacturer’s protocol and luminescence was measured on a FilterMax F3. All experiments were performed in triplicate.

### Immunofluorescence

µ-Slides I^0.4^ Luer (80176; Ibidi) were pre-coated with 0.2% gelatin. Pre-miR transfected HUVEC (1.5×10^6^ cells/mL) were seeded in µ-Slides, incubated for 72 hours at 37°C and 5% CO_2_ in antibiotics-free medium. Cells were fixed with 4% paraformaldehyde for 10 minutes at room temperature. Next, cells were washed three times with PBS, permeabilized with 0.1% Triton X-100 in PBS for 10 minutes and incubated for 1 hour at room temperature in blocking solution: 5% normal goat serum, 5% bovine serum albumin in PBS. Primary antibody incubation (VE-Cadherin, sc-9989; claudin-5, sc-374221; Santa Cruz Biotech) was performed overnight at 4°C. Slides were washed and stained with secondary antibodies (Alexa Fluor 488; 1: 400; Invitrogen). Images of 10 randomly chosen microscopic fields per slide 200x magnification were acquired using a LEICA-TCS SP5 Confocal Microscope (Leica) with the Leica application suite AF software, version 2.7.

### Chemotaxis Assays

One day prior to the experiment, chemotaxis slides (Ibidi) and RPMI media were equilibrated at 37°C and 5% CO_2_ in a humidified incubator. 2x10^6^ Monocytes were resuspended in 175µl RPMI supplemented with 20% FCS, 1% Penicillin/Streptomycin/Glutamine and 1% HEPES. After adding 25µl of Bovine Collagen I pH=7 (Thermo Fisher), the suspension was thoroughly mixed and 6µl were introduced in the µ-Slide Chemotaxis (ibidi). The slide was placed in a cell culture incubator for 1h to ensure proper ECM matrix formation. Immediately before time-lapse microscopy, the reservoirs were loaded with RPMI media alone or RPMI media supplemented with 50ng/ml CCL2, respectively, to create a chemokine concentration gradient. Time-lapse microscopy was performed on a Zeiss Axio Observer Z1 equipped with a gas incubation system (Ibidi). For each channel, three representative fields of vision were chosen and pictures were obtained for 4h (1 picture/minute). Single-cell tracking analysis was performed using ImageJ and the Chemotaxis and Migration Tool (Ibidi).

### Statistics

If not stated otherwise, values shown represent means ± standard error of the mean (SEM). N refers to the number of independent experiments with cells obtained from different donors. *p*-values were calculated using student’s *t*-test or paired *t*-test for all data with normal distribution and Wilcoxon signed rank test otherwise. Statistical analyses were performed using GraphPad Prism 7. *p*-values below 0.05 were considered statistically significant (* *p* < 0.05; ** *p* < 0.01; *** *p* < 0.001).

## Results

### Up-Regulation of miR-125a in Response to Inflammatory Stimuli Strongly Impacts the Transcriptome of Endothelial Cells

MiR-125a is derived from its precursor in two isoforms, miR-125a-3p and miR-125a-5p. While miR-125a-5p is abundantly expressed, its counterpart is barely detectable in most tissues (ratio 509:1) (13, 14). Accordingly, miR-125a-5p is further referred to as miR-125a.

**Figure 1:**
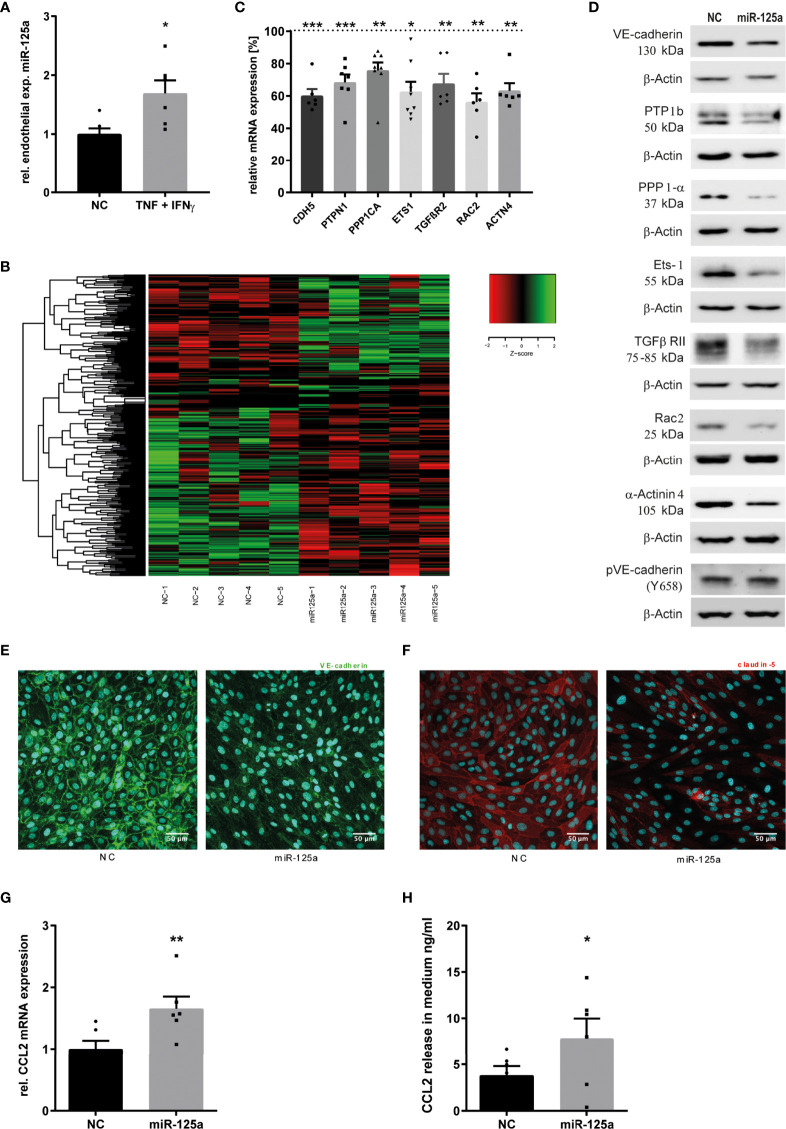
MiR-125a expression upon inflammatory stimulation of HUVEC and its effect on endothelial transcriptome. **(A)** Endothelial expression of miR-125a after cytokine treatment with 25ng/ml TNF and 50 ng/ml IFN-γ for 4 hours (n=6, p<0.05). **(B)** Clustered heatmap of z-scaled expressions of significantly differentially expressed genes in HUVEC transfected with either miR-125a or scrambled control (NC) (Whole transcriptome RNAseq, n=5). Green color indicates enhanced expression, reduced expression is indicated in red color. **(C, D)** mRNA **(**qRT-PCR) and protein (SDS-PAGE) expression of differentially regulated genes extracted from RNAseq data (n=6-8, **p<0.01 ***p<0.001). **(E, F)** Immunofluorescent staining of VE-Cadherin and Claudin-5 in endothelial cells transfected with miR-125a or scrambled control (NC). One exemplary picture of three independent experiments is shown. **(G, H)** expression levels of CCL2 mRNA and protein in HUVEC and cell culture supernatant, respectively (n=6; **p<0.01, *p<0.05).

MiR-125a expression is increased in the blood of patients with acute inflammatory conditions (15–17). Thus, we first investigated whether miR-125a expression in endothelial cells is influenced by inflammatory stimulation. As shown in **Figure 1A**, we detected a sharp increase of miR-125a expression upon cytokine stimulation with tumor necrosis factor (TNF) and interferon-gamma (IFN-γ) (+68.9% ± 22.3%, n=6, *p*<0.05).

To investigate the functional impact of this up-regulation, we analyzed the transcriptome of HUVEC after miR-125a overexpression (transfection efficiency: **Supplementary Figure S3A**) and TNF stimulation. Next Generation Sequencing (NGS) identified 468 genes as differentially regulated (**Figure 1B** and **Supplementary Tables S4**). *In-silico* analysis identified the pathways *GO_regulation of cell adhesion* and *KEGG_adherens junctions* to be significantly regulated by miR-125a (**Supplementary Tables S5, S6**), pointing to a crucial role of miR-125a in inflammation-induced changes of the endothelial barrier.

To identify direct targets of miR-125a possibly regulating endothelial permeability, we combined pathway analysis with *in-silico* target prediction for extracting mRNAs that a) are involved in pathways regulating cell adhesion and cellular junctions and b) contain miR-125a binding sites in their 3’ untranslated region (3’UTR). We found seven potential targets meeting both criteria: VE-Cadherin (CDH5), Protein Tyrosine Phosphatase Non-Receptor Type 1 (PTPN1), Protein Phosphatase 1 Catalytic Subunit Alpha (PPP1CA) ETS proto-oncogene 1 (ETS-1), Transforming Growth Factor Beta Receptor 2 (TGFβR2), Rac Family Small GTPase 2 (RAC2) and Actinin Alpha 4 (ACTN4). Validation of NGS results in HUVEC transfected with miR-125a by qRT-PCR resulted in a significantly decreased mRNA expression of all investigated targets (**Figure 1C**): CDH5 (-39.9% ± 3.8%; n=6; *p*<0.001), PTPN1 (-31.6% ± 4.5%; n=7; *p*<0.001), PPP1CA (-24.6% ± 6.5%; n=8; *p*<0.01), ETS1 (-31.3% ± 7.2%; n=8; *p*<0.05), TGFβR2 (-32.4% ± 5.6%; n=6; *p*<0.01), RAC2 (-43.7% ± 4.9%; n=6; *p*<0.01), ACTN4 (-36.7% ± 4.2%; n=6; *p*<0.01). These results could be confirmed on protein level by western blot analysis for all targets (**Figure 1D**). Following TNF stimulation, endothelial barrier disruption depends on the phosphorylation and subsequent degradation of VE-Cadherin (18). Accordingly, we confirmed in western blot analysis that miR-125a overexpression increased expression of phosphorylated VE-Cadherin (pVE-Cadherin, Tyr658) after TNF stimulation (**Figure 1D**).

**Figure 2:**
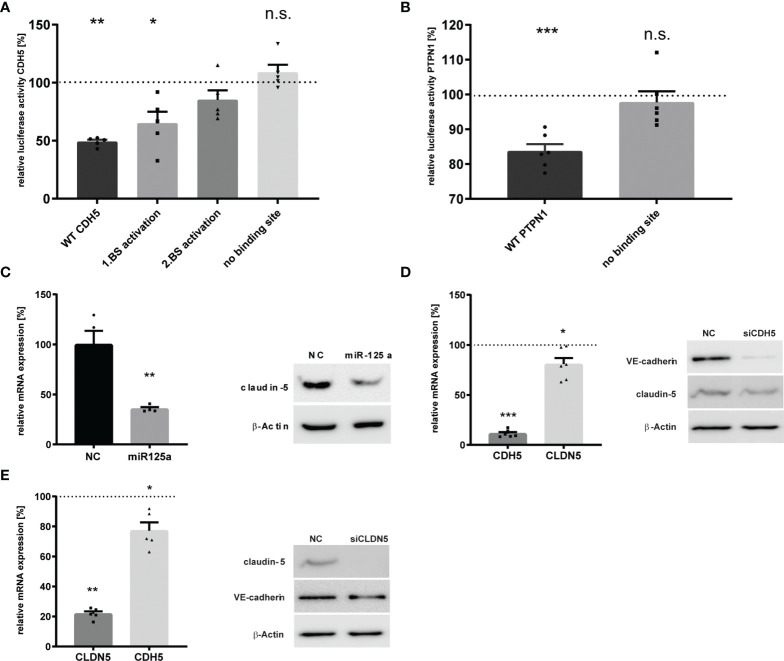
Validation of the miR-125a-related network in HUVEC. **(A)** CDH5 3`UTR Luciferase reporter gene activity after co-transfection of the luciferase constructs with miR-125a or scrambled control (NC). Luciferase constructs: wt_CDH5: both binding sites of miR-125a in the CDH5 3`UTR are functional; mut1_miR-125a: site-directed mutagenesis of binding site 1; mut2_miR-125a: site-directed mutagenesis of binding site 2; mut1+2_miR-125a: site-directed mutagenesis of both binding sites (n=5, p<0.01). **(B)** PTPN1 3`UTR Luciferase reporter gene activity with functional miR-125a binding site (wt PTPN1) and site-directed mutagenesis of the miR-125a binding site (mut1_miR-125a). n=6 p<0.001). **(C)** mRNA (left) and protein (right) expression of Claudin-5 after transfection with miR-125a or scrambled control (NC). n=4, p<0.01. **(D, E)** Knock-down of CDH5 **(D)** and CLDN5 **(E)** by RNA interference. mRNA (left) and protein (right) levels as measured by qRT-PCR and SDS-PAGE, respectively. n=5-6. ***p<0.001, **p<0.01, *p<0.05, n.s., not significant.

Moreover, NGS data showed a strong downregulation of Claudin 5 (CLDN5) expression, although its 3`-UTR does not contain potential miR-125a target sites. This finding was corroborated by qRT-PCR and western blot analysis (**Figure 2C**: qRT-PCR: -64.5% ± 1.5%; n=4; p<0.01; protein: -80.2% ± 7.7%; n=4). Accordingly, not only a reduced surface expression of CDH5 (**Figure 1E**) but also of CLDN5 (**Figure 1F**) was detected by immunofluorescence after transfection of miR-125a in TNF-stimulated HUVEC monolayers. We decided to pursue this hint as CLDN5 has been shown to play an important role in the TNF-mediated disruption of the endothelial barrier (19).

In the analysis of the transcriptome, it was particularly striking that the expression of the CC-chemokine ligand 2 (CCL2) was strongly enhanced upon miR-125a overexpression (1.48-fold). This finding could be validated both by qPCR and ELISA (**Figures 1G, H**: qRT-PCR: -65.6% ± 17.7%; n=6; p<0.01; protein: -89.0% ± 14.1%; n=6; p<0.05). Since CCL2 is one of the most prominent chemokines involved in migration and activation of monocytes, this data suggests miR-125a to be additionally involved in monocyte recruitment during the inflammatory cascade.

Collectively, we found 8 genes with particular importance for endothelial barrier function to be down-regulated, and one major monocyte-attracting chemokine to be up-regulated in response to endothelial miR-125a overexpression.

### CDH5, PTPN1, PPP1CA and ETS1 Are Directly Regulated Targets of miR-125a While CLDN5 Expression Is Regulated Through Mutual CLDN5/CDH5 Regulatory Crosstalk

In order to assess whether the potential targets with in-silico predicted binding sites are indeed directly regulated by miR-125a, we performed luciferase reporter assays containing the *Renilla* luciferase gene fused to the 3’UTR of the respective target. While PPP1CA and ETS-1 were already known and confirmed targets of miR-125a (20, 21), direct interaction of miR-125a with the respective 3’UTR of CDH5, PTPN1, TGFβR2, RAC2 and ACTN4 had to be determined. Transient co-transfection of HEK-293 cells with the respective reporter vectors and miR-125a confirmed repression of the wild-type CDH5 plasmid by 50.9% ± 1.53% (n=5, *p*<0.01; **Figure 2A**). Site-directed mutagenesis of the two predicted binding sites reconstituted luciferase intensity, revealing that both are indeed functional binding sites of miR-125a. Co-transfection of the wild-type PTPN1 plasmid with miR-125a decreased luciferase activity by 16.3% ± 1.86% (n=6, *p*<0.001; **Figure 2B**), and mutation of the binding site restored luciferase activity. In contrast, luciferase activity of the vector with 3’UTR of TGFβR2, RAC2 and ACTN4 did not change after co-transfection with miR-125a (data not shown), indicating that they are not direct targets of miR-125a.

Direct regulation of CLDN5 by miR-125a was unlikely since the 3’UTR of CLDN5 does not contain any putative binding sites. This assumption was confirmed by luciferase reporter assays revealing no significant regulation after co-transfection with miR-125a (data not shown). Recent evidence suggests that expression of CLDN5 strongly depends on CDH5 expression indicating a transcriptional relationship (22, 23). Thus, we hypothesized that miR-125a affects CLDN5 expression indirectly *via* direct targeting of CDH5. To test this, we knocked-down either CDH5 or CLDN5 expression using siRNA and analyzed its effect on the expression of the respective molecule. We could show a reciprocal regulation for both adhesion molecules since knock-down of CDH5 caused a significant downregulation of CLDN5 and vice versa (**Figure 2D**: qRT-PCR: CLDN5 -19.3% ± 5.7%, n=6, *p*<0.05; CDH5 -22.8% ± 4.9%, n=5, *p* < 0.05. **Figure 2E**: protein: CLDN5 -32.4% ± 8.6%; CDH5 -27.8%± 0.3%).

Together, these findings indicate that CDH5 and PTPN1 are newly discovered *bona fide* targets of miR-125a, PPP1CA and ETS-1 are known direct targets, while CLDN5 represents an indirect component of the miR-125a-regulated functional network.

### A miR-125a-Driven Functional Network Induces a Hyperpermeable Phenotype of Endothelial Cells

**Figure 3:**
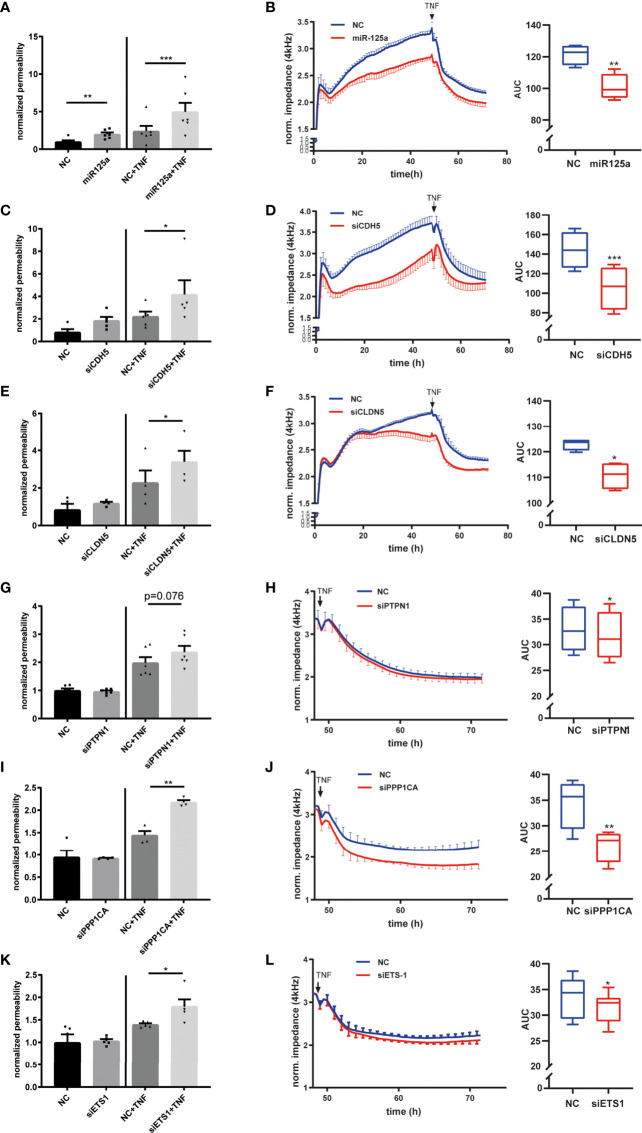
Functional relevance of the miR–125a-driven network on endothelial permeability. **(A)** Transfection of HUVEC with miR-125a increased FITC-Albumin permeability across endothelial monolayers under basal and stimulated (TNF 25ng/ml, 24 h) conditions. FITC-Albumin passage was measured 72 hours after transfection (n=6, ***p<0.001, **p<0.01, *p<0.05). **(B)** Compared to scrambled control (NC, blue), miR-125a (red) impaired endothelial barrier function as indicated by decreased endothelial electrical impedance in both basal and inflammatory conditions mimicked by TNF stimulation (n=4, **p<0.01). **(C–L)** Individual knock-down of each of the functional network members by specific siRNA interference targeting the indicated gene. **(C, E, G, I, K)** Measurement of FITS-BSA-Passage in basal and TNF-stimulated conditions **(D, F, H, J, L)** Measurement of electrical impedance, basal and after TNF stimulation (n=4-6, ***p<0.001, **p<0.01, *p<0.05).

We next aimed at investigating the impact of the identified miR-125a-driven functional network on the endothelial barrier. To this end, we assessed the transendothelial passage of FITC-conjugated albumin and the electric impedance of the endothelial monolayer *via* Electric Cell-Substrate Impedance Sensing (ECIS) in real-time. As shown in **Figure 3A**, we could show that overexpression of miR-125a significantly impaired endothelial barrier function, as shown by an increase in basal and TNF-induced passage of macromolecules (**Figure 3A**: MiR *vs*. NC: 116.0% ± 29.1%, n=6, p<0.01; miR+TNF *vs*. NC+TNF: 128%± 27.6%, n=6, p<0.001). These findings were corroborated by a decrease of the endothelial electrical impedance in HUVEC overexpressing miR-125a (**Figure 3B**: AUC -17.1% ± 4.5%, n=4, p<0.01).

The next step was to investigate whether the detected miR-125a-specific network members indeed account for the hyperpermeable phenotype observed after miR-125a overexpression. We performed siRNA knock-down of CDH5, PTPN1, PPP1CA, ETS1 and CLDN5 (knock-down efficiency: **Supplementary Figure S3C**). Knock-down of CDH5 or CLDN5 increased permeability as measured by FITC-albumin flux and decreased impedance measured by ECIS in basal as well as in TNF-stimulated HUVEC monolayers (**Figures 3C–F**: Permeability assay: siCDH5+TNF *vs*. NC+TNF: 82.4% ± 23.9%, n=5, *p*<0.05; siCLDN5+TNF *vs*. NC+TNF: 61.4% ± 16.2%, n=4, *p*<0.05; ECIS: CDH5: AUC -27.4 ± 4.5%, n=4, *p*<0.01, CLDN5: AUC -17.1% ± 4.5%, n=4, *p*<0.01). Monolayer impedance after TNF stimulation in ECIS was significantly decreased after PTPN1 knock-down. Permeability as measured by albumin-flux was also increased, however, without reaching statistical significance (**Figures 3G, H**: Permeability assay: siPTPN1+TNF *vs*. NC+TNF 16.7 ± 11.2%, n=8, p=0.149; ECIS: AUC after TNF -4.2 ± 1.7%, n= 4, *p*<0.05). Knock-down of PPP1CA or ETS1 did not affect the endothelial barrier function under basal conditions but significantly enhanced barrier breakdown upon 24 hours TNF stimulation in permeability assays and ECIS (**Figures 3I–L**: Permeability assay: PPP1CA+TNF *vs*. NC+TNF: 44.9% ± 10.3%, n=4, *p*<0.01; ETS1+TNF *vs*. NC+TNF: 28.5% ± 9.3%, n=5, *p*<0.05; ECIS: PPP1CA: AUC after TNF -23.8 ± 2.1%, n=4, *p*<0.01; ETS1: AUC after TNF -5.0% ± 3.2%, n=6, *p*<0.05).

**Figure 4:**
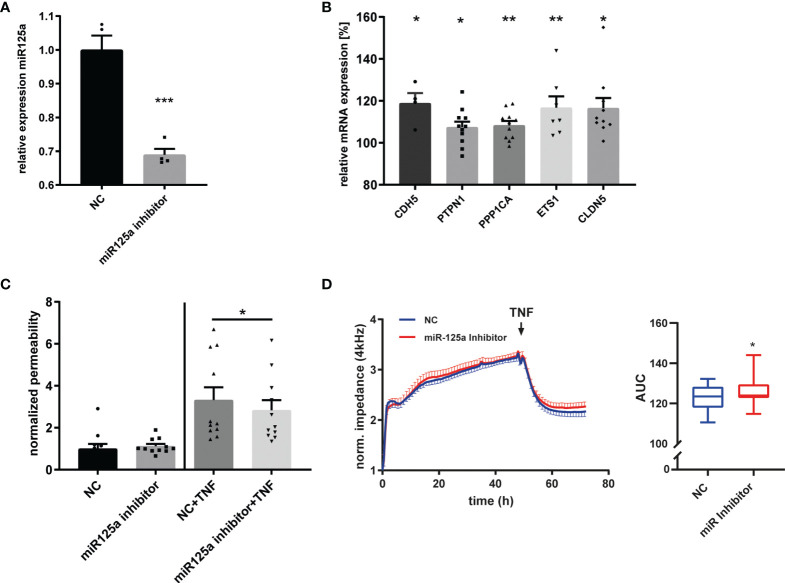
MiR-125a inhibition and its impact on endothelial barrier function. **(A)** Expression of miR-125a in HUVEC after transfection of a miR-125a-inhibitor (“Antimir”) as measured by qRT-PCR (n=4, ***p<0.001). **(B)** mRNA levels of CDH5, PTPN1, PPP1CA, ETS1, CLDN5 after transfection of a miR-125a-inhibitor compared to a scramble control (n=10-11, **p<0.01, *p<0.05). **(C)** Endothelial permeability as measured by FITC-Albumin passage across an endothelial monolayer in basal (left) and inflammatory (right) conditions after transfection of a miR-125a-inhibitor or a scrambled control (NC). n=11, *p<0.05. **(D)** Impedance measurement of the endothelial monolayer after miR-125a inhibition (red) compared to scrambled control (NC, blue). n=11, *p<0.05.

We next transfected HUVEC with a miR-125a-specific miRNA inhibitor (Antimir) which reduced miR-125a expression in qRT-PCR (-31.1% ± 1.5%, n=4, p<0.001; **Figure 4A**), and consecutively increased mRNA levels of the five functional network targets CDH5 (+18.9% ± 4.1%; n=4; *p*< 0.05), PTPN1 (+7.5% ± 2.5%; n=11; *p*<0.05), PPP1CA (+8.4% ± 1.9%; n=11; *p*<0.01), ETS1 (+16.8% ± 5.0%; n=7; *p*<0.01), and CLDN5 (+16.5% ± 4.4%; n=10; *p*<0.05, **Figure 4B**). As expected, miR-125a knock-down was able to attenuate the TNF-induced barrier disruption (**Figure 4C**, albumin flux -10.42% ± 15.14%, n=11, p<0.05), and increased monolayer impedance in ECIS (**Figure 4D**; AUC +2.3% ± 0.9%, n=11, *p*<0.05).

In summary, we discovered a regulatory network driven by miR-125a that regulates the barrier function of human endothelial cells in response to inflammatory stimuli.

### In Primary Human Monocytes, Chemokine Receptor Type 2 Is Regulated by miR-125a

**Figure 5:**
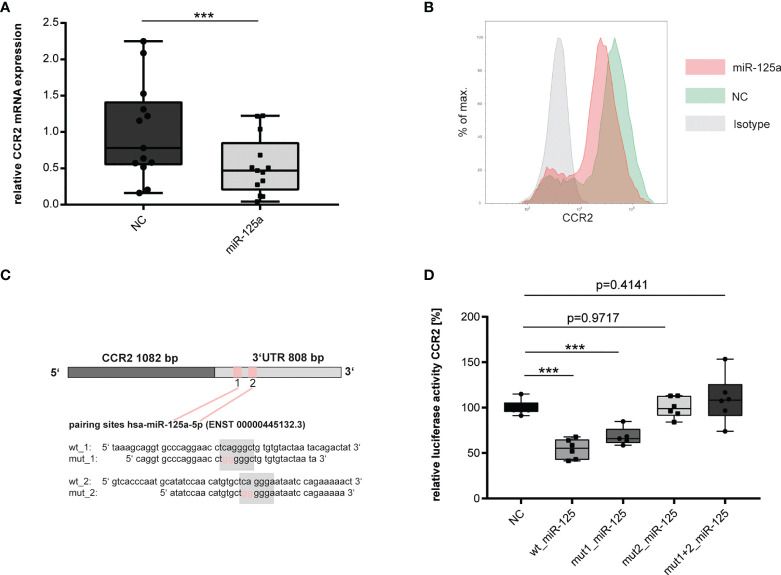
Validation of miR-125a direct target CCR2. **(A)** mRNA (n=13,***p<0.001) and **(B)** protein (n=3, one exemplary experiment is shown) expression of CCR2 in primary human monocytes after tranfection of miR-125a or scrambled control (NC). **(C)** Schematic representation of the two predicted miR-125a binding sites within the 3`UTR of CCR2, including the site-directed oligonucleotide exchanges introduced for luciferase experiments. **(D)** CCR2 3`UTR Luciferase reporter gene activity after co-transfection of the luciferase constructs with miR-125a or scrambled control (NC). Luciferase constructs: wt_CCR2: both binding sites of miR-125a in the CCR2 3`UTR are functional; mut1_miR-125a: site-directed mutagenesis of binding site 1; mut2_miR-125a: site-directed mutagenesis of binding site 2; mut1+2_miR-125a: site-directed mutagenesis of both binding sites (n=6, ***p<0.0001).

In endothelial cells, we found the Chemokine CCL2 to be strongly induced by miR-125a (**Figures 1G, H**). CCL2 is a specific ligand of the C-C Chemokine Receptor Type 2 (CCR2). CCR2, in turn, is strongly expressed on monocytes and is the central mediator of monocyte chemotaxis (24, 25). Sebastiani et al. showed that miR-125a directly regulated CCR2 on regulatory T-cells (26). We hypothesized that miR-125a may also regulate CCR2 on monocytes thus orchestrating the interplay of the endothelium with monocytes during the onset of acute inflammation. To investigate our hypothesis, we transfected primary human monocytes with miR-125a (transfection efficiency: ~61%, **Supplementary Figure S3B**) and analyzed CCR2 expression. As shown in **Figure 5**, overexpression of miR-125a significantly reduced CCR2 mRNA (**Figure 5A**: -46.3% ± 21.5%, n=13, p<0.001) and protein expression (**Figure 5B**).

We next investigated whether miR-125a directly interacts with the 3`UTR of CCR2. *In-silico* analyses revealed two putative seed sequences of miR-125a at positions 113-119 and 169-176 of the 3`UTR of CCR2 (**Figure 5C**). To provide experimental proof that CCR2 is a direct target of miR-125a, we cloned the full 3`UTR of the CCR2 gene into reporter constructs and performed luciferase reporter gene assays. Co-transfection of miR-125a and the reporter construct significantly decreased luciferase activity (**Figure 5D**, -45.6 ± 5.53, p<0.001, n=6). Site-directed mutagenesis of the miR-125a seed sequence 1 partially restored luciferase activity to 68% ( ± 5.42%, n=5, p>0.001), whereas both mutation of seed sequence 2 as well as mutation of seed sequences 1 + 2 completely restored luciferase activity, abolishing the effect of miR-125a in reporter gene quenching (n=6, p=0.818/0.414).

These results corroborate previous data from Sebastiani et al. (26) showing that miR-125a also regulates CCR2 expression in human monocytes by directly interacting with the seed sequences in the CCR2-3`UTR.

### MiR-125a Regulates Monocyte Chemotaxis *via* CCR2

**Figure 6:**
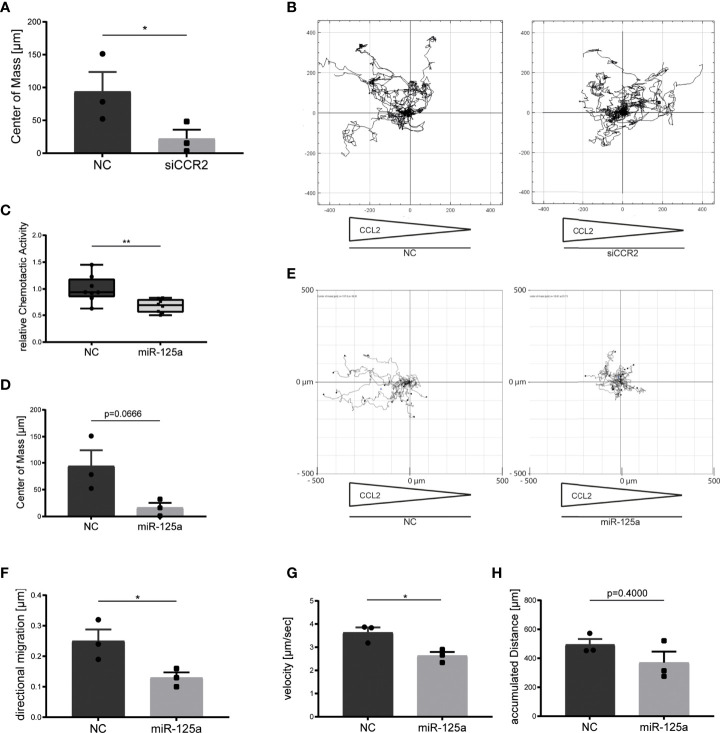
Effect of miR-125a on monocyte chemotaxis. CCL2-specific monocyte chemotaxis after transfection of siRNA specifically targeting CCR2 **(A, B)**, miR-125a **(C–H)** or the respective scrambled controls (NC). Monocyte migration was recorded by time-lapse microscopy and subsequently analyzed by single-cell tracking analyses. (n=3, *p<0.05, **p<0.01). **(A)** Center of mass representing the average of all single cell endpoints. **(B)** Exemplary chemotaxis plot of three independent experiments. Arrows below the plot indicate the CCL2 concentration gradient. **(C, D, F–H)** miR-125a impacts chemotactic activity, center of mass, directional migration and velocity without affecting the accumulated distance. **(E)** Exemplary chemotaxis plot of three independent experiments. Arrows below the plot indicate the CCL2 concentration gradient.

To corroborate the central role of CCR2 for monocyte chemotaxis, we first conducted gene-specific knockdown by RNA interference and combined time-lapse microscopy with subsequent cell tracking analyses. This revealed a clear impairment of CCL2-triggered chemotaxis after CCR2 knockdown (**Figure 6A, B**; -71.6% ± 16.2%, center of mass: Control 93.94 ± 29.64µm; siCCR2: 22.33 ± 13.45µm, p=0.0475, n=3). We next assessed if miR-125a induces a similar phenotype by performing Boyden chamber migration assays and detected a significant impairment of CCL2-directed migration after transfection of miR-125a (**Figure 6C**, -32.2% ± 9.9%, n=8, p<0.01). Accordingly, time-lapse microscopy experiments and subsequent cell-tracking analysis revealed a significant reduction of CCL2-induced directional migration (**Figures 6–F**, -48% ± 13.3%; Control: 0.25 ± 0.038µm; miR-125a: 0.13 ± 0.017µm, n=3, p<0.05) and velocity (**Figure 6G**, -27.5% ± 6.2%; Control: 3.63 ± 0.39µm miR-125a: 2.63 ± 0.28µm, n=3, p<0.05) after miR-125a overexpression without significantly reducing the overall accumulated distance (**Figure 6H**, n=3, p=0.4). These results show that miR-125a inhibits monocyte chemotaxis by specifically targeting CCR2.

### Inflammation Represses miR-125a and Thereby Induces CCR2 in Primary Human Monocytes

**Figure 7:**
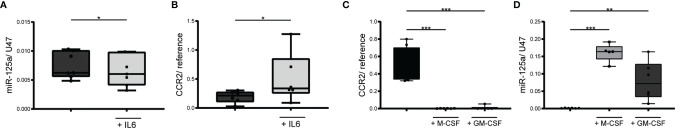
MiR-125a and CCR2 expression in monocytes during inflammation and monocyte differentiation. Expression levels of miR-125a **(A)** and CCR2 **(B)** after incubation of primary human monocytes with IL6 for 3 hours (n=7, *p<0.05). Quantification of CCR2 **(C)** and miR-125a **(D)** expression in primary human monocytes immediately after differentiation with M-/GM-CSF for 6 days. (n=5-6, **p<0.01, ***p<0.001).

We next analyzed whether acute inflammation may not only impact miR-125a expression in endothelial cells but also in human monocytes. To this end, we stimulated primary human monocytes with IL-6 (50ng/ml) and quantified the expressional changes of miR-125a and its target gene CCR2. We found miR-125a to be significantly reduced (**Figure 7A**, -12.7% ± 15,.%, n=7, p<0.05), whereas expression of CCR2 was induced as early as 3 hours after stimulation (**Figure 7B**, +164%, n=6, p<0.05). It thus can be assumed that the increase in migratory capacity of monocytes upon inflammatory stimuli is mediated by down-regulation of miR-125a.

### Expression of miR-125a Strongly Increases During Monocyte-to- Macrophage Differentiation

After crossing the endothelial barrier and reaching the perivascular tissue, monocytes differentiate into macrophages and become resident. Previous studies have shown that the monocyte-to-macrophage differentiation is associated with a sharp decrease of CCR2 expression (27–29). The underlying mechanisms have remained elusive, so far. In accordance with these studies, we found CCR2 to have almost disappeared after inducing macrophage differentiation *in vitro* (**Figure 7C**, M-CSF/GM-CSF >-99% ± 0.2%, n=6, p<0.001). Simultaneously, a dramatic increase in miR-125a expression occurred (**Figure 7D**, M-CSF +11,700% ± 800%, n=5, p<0.001; GM-CSF +5,797% ± 1,665%, n=6, p<0.01), suggesting that induction of miR-125a mediates the loss of CCR2 during monocyte-to-macrophage differentiation, thus contributing to sedentariness.

## Discussion

Extravasation of leukocytes from the bloodstream to the inflamed tissue is a hallmark of the early inflammatory cascade. This dynamic process is characterized by complex interactions between migrating leukocytes and the transiently passable endothelial barrier, which need to be precisely orchestrated to initiate an effective early immune response (30–32). Yet, the underlying regulatory mechanisms are widely unknown.

Increasing evidence proposes an impact of miRNAs on endothelial and immune cell function (33–37). While these studies focused on single miRNA/target interactions, we here aimed to identify a miRNA-driven circuitry that regulates the inflammatory cascade on a cell-type-spanning level. We focused on miR-125a, as recent studies detected elevated levels of miR-125a in the blood of patients with acute inflammatory conditions associated with endothelial dysfunction (15–17).

We first focused on the impact of miR-125a on endothelial cells. Using NGS-based transcriptome analysis after miR-125a overexpression of HUVEC cells, we could identify the pathways *GO_regulation of cell adhesion* and *KEGG_adherens junctions* to be specifically affected, which led us to assume miR-125a to be involved in the regulation of endothelial cell permeability. Indeed, functional analyses showed that miR-125a overexpression induced a hyperpermeable phenotype during the acute inflammatory response. Both NGS and functional data thus clearly indicated that miR-125a has barrier-disruptive properties. To find the underlying mechanism, we extracted genes from the NGS dataset that displayed a) a strong endothelial expression and b) differential regulation after overexpression of miR-125a. We were able to newly elucidate a complex regulatory network directed by miR-125a, consisting of the two adhesion molecules VE-Cadherin (CDH5) and Claudin-5 (CLDN5), of two regulatory tyrosine phosphatases (PTPN1, PPP1CA) and of the transcription factor ETS-1. Individual knockdown of each of the network targets independently increased endothelial permeability. Central effector element in this network is CDH5, the central gatekeeper of endothelial barrier function and regulator of paracellular permeability (38, 39). The other direct target genes evaluated in this study, ETS-1, PTPN1 and PPP1CA, exert their effects *via* CDH5 thus strongly multiplying the impact of miR-125a:

ETS-1 exhibits barrier-protective effects *via* transcriptional induction of VE-Cadherin (40, 41). MiR-125a-mediated repression of ETS-1 reduces VE-Cadherin levels and further increases endothelial barrier permeability during acute inflammation. The two phosphatases, on the other hand, act on a post-transcriptional level. Posttranslational phosphorylation of VE-Cadherin at different tyrosine residues and subsequent internalization is one of the main triggers for endothelial barrier hyperpermeability during acute inflammation and transendothelial leukocyte migration (18, 42). PTPN1, coding for protein phosphatase 1B, and PPP1CA, a subunit of the protein phosphatase 1, have been shown to prevent phosphorylation and subsequent internalization of VE-Cadherin, thus stabilizing adherens junctions and dampening endothelial barrier dysfunction during acute inflammation (43–46). As expected, targeting of these two phosphatases by miR-125a resulted in increased levels of phospho-VE-Cadherin (Tyr658) and increased permeability of the endothelial monolayer. Phosphorylation at this tyrosine residue Tyr658 is required for leukocyte transendothelial migration (47) and inflammatory triggered hyperpermeability (18). The second adhesion molecule significantly down-regulated by miR-125a, CLDN5, did not reveal as a direct target gene. In fact, we could show that it is indirectly regulated by CDH5. We could identify a secondary negative feedback loop: CDH5 down-regulation leads to CLDN5 repression, which then further decreases CDH5 expression, thereby amplifying the regulatory impact of miR-125a. The mechanisms underlying the CDH5-CLDN5 loop have already been described by Morini et al. (22).

Increased endothelial permeability is associated with enhanced leukocyte transmigration through the endothelial monolayer (42, 47). Guided by the surprising finding of our NGS analysis that expression levels of the chemokine CCL2 in stimulated endothelial cells after miR-125a transfection were strongly increased, we next turned our attention to monocytes as the master mediator of monocyte trafficking is CCL2. Produced by endothelia, it is recognized by the chemotactic receptor CCR2, abundantly expressed on nearly all classical monocytes, that subsequently prompts them to actively migrate towards the inflammatory focus (27, 48, 49). We hypothesized that miR-125a, which is also expressed in monocytes (33, 50), might modulate the CCL2/CCR2 axis in these immune cells thereby regulating their trafficking under inflammatory conditions. We identified two novel binding sites of miR-125a within the 3`UTR of CCR2. We further could show that overexpression of miR-125a down-regulated CCR2 expression levels and strongly impaired CCL2-specific monocyte chemotaxis. Under inflammatory conditions, miR-125a expression in primary human monocytes was strongly diminished and CCR2 was concomitantly induced, enabling increased monocyte trafficking to the inflammatory site.

After entering the inflamed area, monocytes differentiate into macrophages, terminate CCR2 expression, and become stationary (51–53). The molecular mechanisms underlying this CCR2-specific downregulation, however, remain elusive so far. We here provide novel evidence that miR-125a is dramatically induced upon stimulation of monocytes with macrophage-inducing differentiation factors (M-CSF, GM-CSF), while the CCR2 almost completely disappears. We thus propose that direct interaction of miR-125a with the 3`UTR of CCR2 is a previously underestimated molecular mechanism that regulates macrophage trafficking and residence.

**Figure 8:**
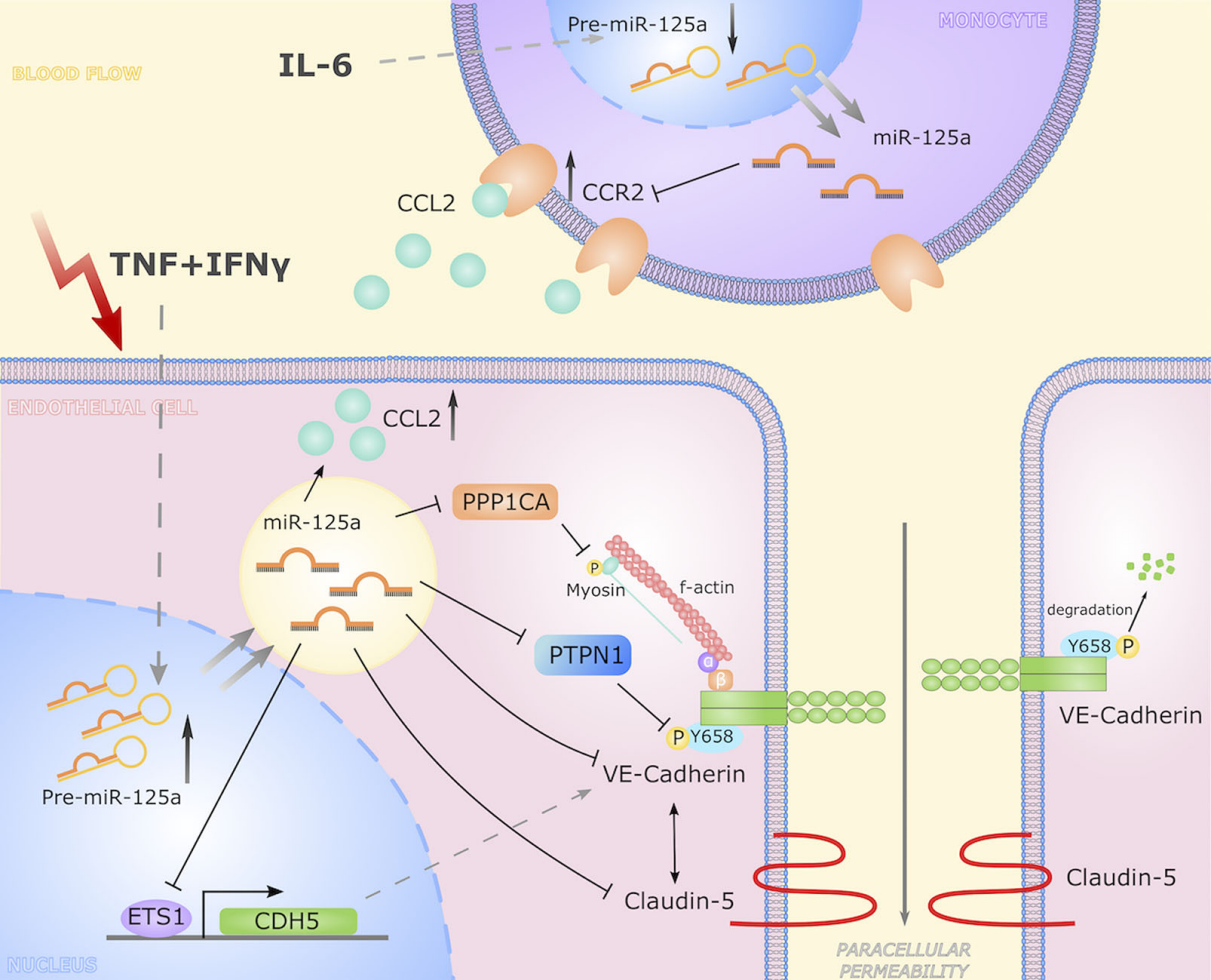
Graphical representation of the miR-125a driven cell-crossing functional network. In response to inflammatory stimulation, the expression of miR-125a increases in endothelial cells. Here, miR-125a directly regulates CDH5, PTPN1, PPP1CA, and ETS-1 while CLDN5 levels are reciprocally linked to CDH5 expression. By reducing expression of ETS-1, miR-125a inhibits VE-Cadherin transcription. *Via* targeting PTPN1 and PPP1CA, miR-125a affects levels of phosphorylated VE-Cadherin, destabilizing adherens junctions and thus increasing paracellular permeability. In turn, monocytic expression of miR-125a is reduced upon inflammatory stimulation, leading to an induction of CCR2 expression, impacting monocyte chemotaxis.

In summary, we discovered and characterized a novel regulatory network directed by miR-125a that regulates and synchronizes the process of endothelial barrier permeability and monocyte migration upon inflammatory stimulation. While previous studies already identified cell-type spanning effects of miRNAs *via* shuttling of extracellular vesicles between cell types (54–57), we here report that one miRNA, although disparately regulated in endothelia and monocytes, directs a biologic process in a synergistic and mutually reinforcing manner (illustrated in **Figure 8**). These findings expand the knowledge on regulation of inflammatory processes and may open up new roads in diagnosis and therapy of inflammation-driven diseases.

## Data Availability Statement

The datasets of RNA-Seq presented in this study can be found in online repositories: https://www.ncbi. nlm.nih.gov/geo/, GSE196161. Further inquiries can be directed to the corresponding author.

## Ethics Statement

The studies involving human participants were reviewed and approved by ethics committee of the medical faculty of the Ludwig-Maximilians-University München (LMU), Germany. The participants provided their written informed consent to participate in this study.

## Author Contributions

MBM and MH designed and performed experiments, analyzed and interpreted data and wrote the manuscript. LL, ST, DE, VL, and KP performed experiments and analyzed and interpreted data. SH performed experiments, analyzed data and prepared figures. MS provided critical tools and edited the manuscript. SK designed experiments, interpreted data, wrote the manuscript and supervised the study. All authors contributed to the article and approved the submitted version.

## Funding

Funded by the Comprehensive Pneumology Center Munich (82DZL033A2, SK), the Deutsche Forschungsgemeinschaft (DFG, German Research Foundation) - 413635475, the Munich Clinician Scientist Program (MCSP) of the LMU Munich (MBM and MH), and institutional grants of the LMU Munich (Promotionsstudiengang “Molekulare Medizin”, Project Number M37, SK).

## Conflict of Interest

The authors declare that the research was conducted in the absence of any commercial or financial relationships that could be construed as a potential conflict of interest.

## Publisher’s Note

All claims expressed in this article are solely those of the authors and do not necessarily represent those of their affiliated organizations, or those of the publisher, the editors and the reviewers. Any product that may be evaluated in this article, or claim that may be made by its manufacturer, is not guaranteed or endorsed by the publisher.

## References

[1] Hotchkiss RS Monneret G Payen D. Sepsis-Induced Immunosuppression: From Cellular Dysfunctions to Immunotherapy. Nat Rev Immunol (2013) 13:862–74. doi: 10.1038/nri3552 PMC4077177 24232462

[2] Opal SM van der Poll T. Endothelial Barrier Dysfunction in Septic Shock. J Intern Med (2015) 277:277–93. doi: 10.1111/joim.12331 25418337

[3] Chatterjee V Beard RS Jr. Reynolds JJ Haines R Guo M Rubin M. MicroRNA-147b Regulates Vascular Endothelial Barrier Function by Targeting ADAM15 Expression. PLoS One (2014) 9:e110286. doi: 10.1371/journal.pone.0110286 25333931 PMC4198252

[4] Rajput C Tauseef M Farazuddin M Yazbeck P Amin MR Avin Br V. MicroRNA-150 Suppression of Angiopoetin-2 Generation and Signaling Is Crucial for Resolving Vascular Injury. Arterioscler Thromb Vasc Biol (2016) 36:380–8. doi: 10.1161/ATVBAHA.115.306997 PMC4732888 26743170

[5] Yang B Huang X Xu S Li L Wu W Dai Y. : Decreased miR-4512 Levels in Monocytes and Macrophages of Individuals With Systemic Lupus Erythematosus Contribute to Innate Immune Activation and Neutrsophil NETosis by Targeting TLR4 and CXCL2. Front Immunol (2021) 12:756825:756825. doi: 10.3389/fimmu.2021.756825 34721432 PMC8552026

[6] Chen K Rajewsky N. The Evolution of Gene Regulation by Transcription Factors and microRNAs. Nat Rev Genet (2007) 8:93–103. doi: 10.1038/nrg1990 17230196

[7] Tanaka T Narazaki M Kishimoto T. IL-6 in Inflammation, Immunity, and Disease. Cold Spring Harb Perspect Biol (2014) 6:a016295. doi: 10.1101/cshperspect.a016295 25190079 PMC4176007

[8] Clahsen T Schaper F. Interleukin-6 Acts in the Fashion of a Classical Chemokine on Monocytic Cells by Inducing Integrin Activation, Cell Adhesion, Actin Polymerization, Chemotaxis, and Transmigration. J Leukoc Biol (2008) 84:1521–9. doi: 10.1189/jlb.0308178 18765478

[9] Lopez-Ramirez MA Wu D Pryce G Simpson JE Reijerkerk A King-Robson J. MicroRNA-155 Negatively Affects Blood-Brain Barrier Function During Neuroinflammation. FASEB J (2014) 28:2551–65. doi: 10.1096/fj.13-248880 24604078

[10] Yee D Shah KM Coles MC Sharp TV Lagos D. MicroRNA-155 Induction *via* TNF-Alpha and IFN-Gamma Suppresses Expression of Programmed Death Ligand-1 (PD-L1) in Human Primary Cells. J Biol Chem (2017) 292:20683–93. doi: 10.1074/jbc.M117.809053 PMC5733604 29066622

[11] Hubner M Hinske CL Effinger D Wu T Thon N Kreth FW. Intronic miR-744 Inhibits Glioblastoma Migration by Functionally Antagonizing Its Host Gene Map2k4. Cancers (2018) 10(11):400. doi: 10.3390/cancers10110400 PMC6266622 30366472

[12] Hirschberger S Hubner M Strauss G Effinger D Bauer M Weis S. Identification of Suitable Controls for miRNA Quantification in T-Cells and Whole Blood Cells in Sepsis. Sci Rep (2019) 9:15735. doi: 10.1038/s41598-019-51782-w 31672997 PMC6823537

[13] Kozomara A Griffiths-Jones S. Mirbase: Annotating High Confidence microRNAs Using Deep Sequencing Data. Nucleic Acids Res (2014) 42:D68–73. doi: 10.1093/nar/gkt1181 PMC3965103 24275495

[14] Hinske LC Franca GS Torres HA Ohara DT Lopes-Ramos CM Heyn J. miRIAD-Integrating microRNA Inter- and Intragenic Data. Database (2014) 2014:1–9. doi: 10.1093/database/bau099 PMC4186326 25288656

[15] Li S Zhao D Cui J Wang L Ma X Li Y. Correlation of microRNA-125a/B With Acute Respiratory Distress Syndrome Risk and Prognosis in Sepsis Patients. J Clin Lab Anal (2020) 34:e23098. doi: 10.1002/jcla.23098 31967348 PMC7083491

[16] Tiedt S Prestel M Malik R Schieferdecker N Duering M Kautzky V. RNA-Seq Identifies Circulating miR-125a-5p, miR-125b-5p, and miR-143-3p as Potential Biomarkers for Acute Ischemic Stroke. Circ Res (2017) 121:970–80. doi: 10.1161/CIRCRESAHA.117.311572 28724745

[17] Zhao D Li S Cui J Wang L Ma X Li Y. Plasma miR-125a and miR-125b in Sepsis: Correlation With Disease Risk, Inflammation, Severity, and Prognosis. J Clin Lab Anal (2020) 34:e23036. doi: 10.1002/jcla.23036 32077163 PMC7031612

[18] Orsenigo F Giampietro C Ferrari A Corada M Galaup A Sigismund S. Phosphorylation of VE-Cadherin is Modulated by Haemodynamic Forces and Contributes to the Regulation of Vascular Permeability In Vivo. Nat Commun (2012) 3:1208. doi: 10.1038/ncomms2199 23169049 PMC3514492

[19] Clark PR Kim RK Pober JS Kluger MS. Tumor Necrosis Factor Disrupts Claudin-5 Endothelial Tight Junction Barriers in Two Distinct NF-kappaB-Dependent Phases. PLoS One (2015) 10:e0120075. doi: 10.1371/journal.pone.0120075 25816133 PMC4376850

[20] Ge Y Sun M Wu W Ma C Zhang C He C. MicroRNA-125a Suppresses Intestinal Mucosal Inflammation Through Targeting ETS-1 in Patients With Inflammatory Bowel Diseases. J Autoimmun (2019) 101:109–20. doi: 10.1016/j.jaut.2019.04.014 31014918

[21] Guo S Bai H Megyola CM Halene S Krause DS Scadden DT. Complex Oncogene Dependence in microRNA-125a-Induced Myeloproliferative Neoplasms. Proc Natl Acad Sci U S A (2012) 109:16636–41. doi: 10.1073/pnas.1213196109 PMC3478612 23012470

[22] Morini MF Giampietro C Corada M Pisati F Lavarone E Cunha SI. VE-Cadherin-Mediated Epigenetic Regulation of Endothelial Gene Expression. Circ Res (2018) 122:231–45. doi: 10.1161/CIRCRESAHA.117.312392 PMC5771688 29233846

[23] Taddei A Giampietro C Conti A Orsenigo F Breviario F Pirazzoli V. Endothelial Adherens Junctions Control Tight Junctions by VE-Cadherin-Mediated Upregulation of Claudin-5. Nat Cell Biol (2008) 10:923–34. doi: 10.1038/ncb1752 18604199

[24] Charo IF Ransohoff RM. The Many Roles of Chemokines and Chemokine Receptors in Inflammation. N Engl J Med (2006) 354:610–21. doi: 10.1056/NEJMra052723 16467548

[25] Leuschner F Dutta P Gorbatov R Novobrantseva TI Donahoe JS Courties G. Therapeutic siRNA Silencing in Inflammatory Monocytes in Mice. Nat Biotechnol (2011) 29:1005–10. doi: 10.1038/nbt.1989 PMC3212614 21983520

[26] Sebastiani G Ventriglia G Stabilini A Socci C Morsiani C Laurenzi A. Regulatory T-Cells From Pancreatic Lymphnodes of Patients With Type-1 Diabetes Express Increased Levels of microRNA miR-125a-5p That Limits CCR2 Expression. Sci Rep (2017) 7:6897. doi: 10.1038/s41598-017-07172-1 28761107 PMC5537269

[27] Fantuzzi L Borghi P Ciolli V Pavlakis G Belardelli F Gessani S. Loss of CCR2 Expression and Functional Response to Monocyte Chemotactic Protein (MCP-1) During the Differentiation of Human Monocytes: Role of Secreted MCP-1 in the Regulation of the Chemotactic Response. Blood (1999) 94:875–83. doi: 10.1182/blood.V94.3.875.415k28_875_883 10419877

[28] Ruytinx P Proost P Van Damme J Struyf S. Chemokine-Induced Macrophage Polarization in Inflammatory Conditions. Front Immunol (2018) 9:1930. doi: 10.3389/fimmu.2018.01930 30245686 PMC6137099

[29] Shi C Pamer EG. Monocyte Recruitment During Infection and Inflammation. Nat Rev Immunol (2011) 11:762–74. doi: 10.1038/nri3070 PMC3947780 21984070

[30] Fullerton JN Gilroy DW. Resolution of Inflammation: A New Therapeutic Frontier. Nat Rev Drug Discov (2016) 15:551–67. doi: 10.1038/nrd.2016.39 27020098

[31] Nourshargh S Alon R. Leukocyte Migration Into Inflamed Tissues. Immunity (2014) 41:694–707. doi: 10.1016/j.immuni.2014.10.008 25517612

[32] Vestweber D. How Leukocytes Cross the Vascular Endothelium. Nat Rev Immunol (2015) 15:692–704. doi: 10.1038/nri3908 26471775

[33] Schulert GS Fall N Harley JB Shen N Lovell DJ Thornton S. Monocyte MicroRNA Expression in Active Systemic Juvenile Idiopathic Arthritis Implicates MicroRNA-125a-5p in Polarized Monocyte Phenotypes. Arthritis Rheumatol (2016) 68:2300–13. doi: 10.1002/art.39694 PMC5001902 27014994

[34] Svensson D Gidlof O Turczynska KM Erlinge D Albinsson S Nilsson BO. Inhibition of microRNA-125a Promotes Human Endothelial Cell Proliferation and Viability Through an Antiapoptotic Mechanism. J Vasc Res (2014) 51:239–45. doi: 10.1159/000365551 25116893

[35] Wade SM Ohnesorge N McLoughlin H Biniecka M Carter SP Trenkman M. Dysregulated miR-125a Promotes Angiogenesis Through Enhanced Glycolysis. EBioMedicine (2019) 47:402–13. doi: 10.1016/j.ebiom.2019.08.043 PMC6796559 31466915

[36] Wang J Nie Z Zhao H Gao K Cao Y. MiRNA-125a-5p Attenuates Blood-Spinal Cord Barrier Permeability Under Hypoxia In Vitro. Biotechnol Lett (2020) 42:25–34. doi: 10.1007/s10529-019-02753-8 31696327

[37] Young JA Ting KK Li J Moller T Dunn L Lu Y. Regulation of Vascular Leak and Recovery From Ischemic Injury by General and VE-Cadherin-Restricted miRNA Antagonists of miR-27. Blood (2013) 122:2911–9. doi: 10.1182/blood-2012-12-473017 24009229

[38] Hofmann S Grasberger H Jung P Bidlingmaier M Vlotides J Janssen OE. The Tumour Necrosis Factor-Alpha Induced Vascular Permeability is Associated With a Reduction of VE-Cadherin Expression. Eur J Med Res (2002) 7:171–6. 12010652

[39] Komarova YA Kruse K Mehta D Malik AB. Protein Interactions at Endothelial Junctions and Signaling Mechanisms Regulating Endothelial Permeability. Circ Res (2017) 120:179–206. doi: 10.1161/CIRCRESAHA.116.306534 28057793 PMC5225667

[40] Colas-Algora N Garcia-Weber D Cacho-Navas C Barroso S Caballero A Ribas C. Compensatory Increase of VE-Cadherin Expression Through ETS1 Regulates Endothelial Barrier Function in Response to TNFalpha. Cell Mol Life Sci (2020) 77:2125–40. doi: 10.1007/s00018-019-03260-9 PMC11105044 31396656

[41] Lelievre E Mattot V Huber P Vandenbunder B Soncin F. ETS1 Lowers Capillary Endothelial Cell Density at Confluence and Induces the Expression of VE-Cadherin. Oncogene (2000) 19:2438–46. doi: 10.1038/sj.onc.1203563 10828886

[42] Wessel F Winderlich M Holm M Frye M Rivera-Galdos R Vockel M. Leukocyte Extravasation and Vascular Permeability are Each Controlled *In Vivo* by Different Tyrosine Residues of VE-Cadherin. Nat Immunol (2014) 15:223–30. doi: 10.1038/ni.2824 24487320

[43] Grinnell KL Chichger H Braza J Duong H Harrington EO. Protection Against LPS-Induced Pulmonary Edema Through the Attenuation of Protein Tyrosine Phosphatase-1B Oxidation. Am J Respir Cell Mol Biol (2012) 46:623–32. doi: 10.1165/rcmb.2011-0271OC PMC3359908 22180868

[44] Nakamura Y Patrushev N Inomata H Mehta D Urao N Kim HW. Role of Protein Tyrosine Phosphatase 1B in Vascular Endothelial Growth Factor Signaling and Cell-Cell Adhesions in Endothelial Cells. Circ Res (2008) 102:1182–91. doi: 10.1161/CIRCRESAHA.107.167080 PMC2737681 18451337

[45] Quan X Liu X Qin X Wang Y Sun T Li Z. The Role of LR-TIMAP/PP1c Complex in the Occurrence and Development of No-Reflow. EBioMedicine (2021) 65:103251. doi: 10.1016/j.ebiom.2021.103251 33639401 PMC7921471

[46] Adam AP. Regulation of Endothelial Adherens Junctions by Tyrosine Phosphorylation. Mediators Inflamm (2015) 2015:272858. doi: 10.1155/2015/272858 26556953 PMC4628659

[47] Allingham MJ van Buul JD Burridge K. ICAM-1-Mediated, Src- and Pyk2-Dependent Vascular Endothelial Cadherin Tyrosine Phosphorylation is Required for Leukocyte Transendothelial Migration. J Immunol (2007) 179:4053–64. doi: 10.4049/jimmunol.179.6.4053 17785844

[48] Franca CN Izar MCO Hortencio MNS do Amaral JB Ferreira CES Tuleta ID. Monocyte Subtypes and the CCR2 Chemokine Receptor in Cardiovascular Disease. Clin Sci (Lond) (2017) 131:1215–24. doi: 10.1042/CS20170009 28566450

[49] Gschwandtner M Derler R Midwood KS. More Than Just Attractive: How CCL2 Influences Myeloid Cell Behavior Beyond Chemotaxis. Front Immunol (2019) 10:2759. doi: 10.3389/fimmu.2019.02759 31921102 PMC6923224

[50] Hurst SM Wilkinson TS McLoughlin RM Jones S Horiuchi S Yamamoto N. Il-6 and its Soluble Receptor Orchestrate a Temporal Switch in the Pattern of Leukocyte Recruitment Seen During Acute Inflammation. Immunity (2001) 14:705–14. doi: 10.1016/s1074-7613(01)00151-0 11420041

[51] Phillips RJ Lutz M Premack B. Differential Signaling Mechanisms Regulate Expression of CC Chemokine Receptor-2 During Monocyte Maturation. J Inflammation (Lond) (2005) 2:14. doi: 10.1186/1476-9255-2-14 PMC1308851 16259633

[52] Watanabe S Alexander M Misharin AV Budinger GRS. The Role of Macrophages in the Resolution of Inflammation. J Clin Invest (2019) 129:2619–28. doi: 10.1172/JCI124615 PMC6597225 31107246

[53] Xiong H Carter RA Leiner IM Tang YW Chen L Kreiswirth BN. Distinct Contributions of Neutrophils and CCR2+ Monocytes to Pulmonary Clearance of Different Klebsiella Pneumoniae Strains. Infect Immun (2015) 83:3418–27. doi: 10.1128/IAI.00678-15 PMC4534658 26056382

[54] Liang X Zhang L Wang S Han Q Zhao RC. Exosomes Secreted by Mesenchymal Stem Cells Promote Endothelial Cell Angiogenesis by Transferring miR-125a. J Cell Sci (2016) 129:2182–9. doi: 10.1242/jcs.170373 27252357

[55] Pan Q Ma C Wang Y Wang J Zheng J Du D. Microvesicles-Mediated Communication Between Endothelial Cells Modulates, Endothelial Survival, and Angiogenic Function *via* Transferring of miR-125a-5p. J Cell Biochem (2019) 120:3160–72. doi: 10.1002/jcb.27581 30272818

[56] Wang Y Bai Y Liu Y Wilfried Noel S Yan Q Pham Thi H. Plasma Exosomal miRNAs Involved in Endothelial Injury in Microscopic Polyangiitis Patients. FASEB J (2020) 34:6215–28. doi: 10.1096/fj.201902964R 32232900

[57] Zhou W Fong MY Min Y Somlo G Liu L Palomares MR. Cancer-Secreted miR-105 Destroys Vascular Endothelial Barriers to Promote Metastasis. Cancer Cell (2014) 25:501–15. doi: 10.1016/j.ccr.2014.03.007 PMC4016197 24735924

