# Supplementary material for: Cell-Crossing Functional Network Driven by microRNA-125a Regulates Endothelial Permeability and Monocyte Trafficking in Acute Inflammation
Source: Front Immunol. 2022 Mar 24;13:826047. doi: 10.3389/fimmu.2022.826047 (PMC8986987; doi:10.3389/fimmu.2022.826047)
Supplement: Supplementary file 1 [file DataSheet_1.zip › Supplementary Material/Supplementary Table S1.DOCX]

Supplementary Material

**Table S1.** Primer sequences for qRT-PCR.

| **Target Name** | **UPL Probe Number** | **Primer Sequence** |
| --- | --- | --- |
| TBP | 87 | forward: 5’-GAACATCATGGATCAGAACAACA-3’ |
|  |  | reverse: 5’-ATAGGGATTCCGGGAGTCAT-3’ |
| GAPDH | 60 | forward: 5’-AGCCACATCGCTCAGACAC-3’ |
|  |  | reverse:5’-GCCCAATACGACCAAATCC-3’ |
| CDH5 | 58 | forward: 5’-AAGCCTCTGATTGGCACAGT-3’ |
|  |  | reverse:5’-CTGGCCCTTGTCACTGGT-3’ |
| PTPN1 | 32 | forward: 5’-CGGTCACTTTTGGGAGATG-3’ |
|  |  | reverse: 5’-GCCAGTATTGTGCGCATTT-3’ |
| PPP1CA | 40 | forward: 5’-GACAGCGAGAAGCTCAACCT-3’ |
|  |  | reverse: 5’-CGCGGATCTCGTTCTCTG-3’ |
| ETS1 | 3 | forward: 5’-GCAGAATGAGCTACTTTGTGGA-3’ |
|  |  | reverse: 5’-TTGCTAGGTCCTTGCCTCAC-3’ |
| TGFBR2 | 7 | forward: 5’-GGGAAATGACATCTCGCTGTA-3’ |
|  |  | reverse: 5’-CACCTTGGAACCAAATGGAG-3’ |
| RAC2 | 77 | forward: 5’-GATGCAGGCCATCAAGTGT-3’ |
|  |  | reverse: 5’-CTGATGAGAAGGCAGGTCTTG-3’ |
| ACTN4 | 42 | forward: 5’-AAATACCTCGACATCCCCAAG-3’ |
|  |  | reverse: 5’-GGTCATTATGGCCTTCTCG-3’ |
| CLDN5 | 19 | forward: 5’-CCATGGGATGAGAGAGACAGT-3’ |
|  |  | reverse: 5’-GGCCCTTTCTCGCACTCT-3’ |
| CCR2 | 56 | forward: 5’-TGAGACAAGCCACAAGCTGA-3’ |
|  |  | reverse: 5’-TTCTGATAAACCGAGAACGAGAT-3’ |
| B2M | 42 | forward: 5’-TTCTGGCCTGGAGGCTATC-3’ |
|  |  | reverse: 5’-TCAGAAATTTGACTTTCCATTC-3’ |
