# Supplementary material for: Cell-Crossing Functional Network Driven by microRNA-125a Regulates Endothelial Permeability and Monocyte Trafficking in Acute Inflammation
Source: Front Immunol. 2022 Mar 24;13:826047. doi: 10.3389/fimmu.2022.826047 (PMC8986987; doi:10.3389/fimmu.2022.826047)
Supplement: Supplementary file 1 [file DataSheet_1.zip › Supplementary Material/Supplementary Table S2.DOCX]

Supplementary Material

**Table S2.** Primer sequences for molecular cloning.

| **Gene** | **Primer Sequence** | |
| --- | --- | --- |
| CDH5 | wild-type | forward: 5’-CTCGAGAGGATTCTCTGCAGCCCATT -3’ |
|  |  | reverse: 5’-GCGGCCGCCATGACGAAGGGTGAGCTTG-3’ |
|  | 1st binding site mutated | forward: 5’-ACTGAACCACATTCACAGAAATGGCTTATTA-3’ |
|  |  | reverse: 5’- TAATAAGCCATTTCTGTGAATGTGGTTCAGT-3’ |
|  | 2nd binding site mutated | forward: 5’- TGTTGTCACATCTCACAGAACTGACCCTCAG-3’ |
|  |  | reverse: 5’- CTGAGGGTCAGTTCTGTGAGATGTGACAACA-3’ |
| PTPN1 | wild-type | forward: 5’- GGTACGCTCGAGTGGTGGGAACATTCGAGGTG-3’ |
|  |  | reverse: 5’-  GGATATGCGGCCGCGGCAAAGCGTCAATTTGGGA-3’ |
|  | binding site mutated | forward: 5’- GGGGGGGAGTGTCTCACAGTCTTCTGTGACC-3’ |
|  |  | reverse: 5’- GGTCACAGAAGACTGTGAGACACTCCCCCCC-3’ |
| CCR2 | wild-type | reverse: 5’- ctcgagacgagaagaagaggcataggg-3’ |
|  |  | reverse: 5’- gtttaaacacctttgtctttgtccaggc-3’ |
|  | 1st binding site mutated | forward: 5’-CAGGTGCCCAGGAACCTGGGGGCTGTGTGTACTAAATA-3’ |
|  |  | reverse: 5’-  TATTAGTACACACAGCCCCCAGGTTCCTGGGCACCTG-3’ |
|  | 2nd binding site mutated | forward: 5’-  ATATCCAACATGTGCTGGGGGAATAATCCAGAAAAA-3’  reverse: 5’-  TTTTTCTGGATTATTCCCCCAGCACATGTTGGATAT-3’ |
