# Supplementary figures and images for: Cell-Crossing Functional Network Driven by microRNA-125a Regulates Endothelial Permeability and Monocyte Trafficking in Acute Inflammation
Source: Front Immunol. 2022 Mar 24;13:826047. doi: 10.3389/fimmu.2022.826047 (PMC8986987; doi:10.3389/fimmu.2022.826047)

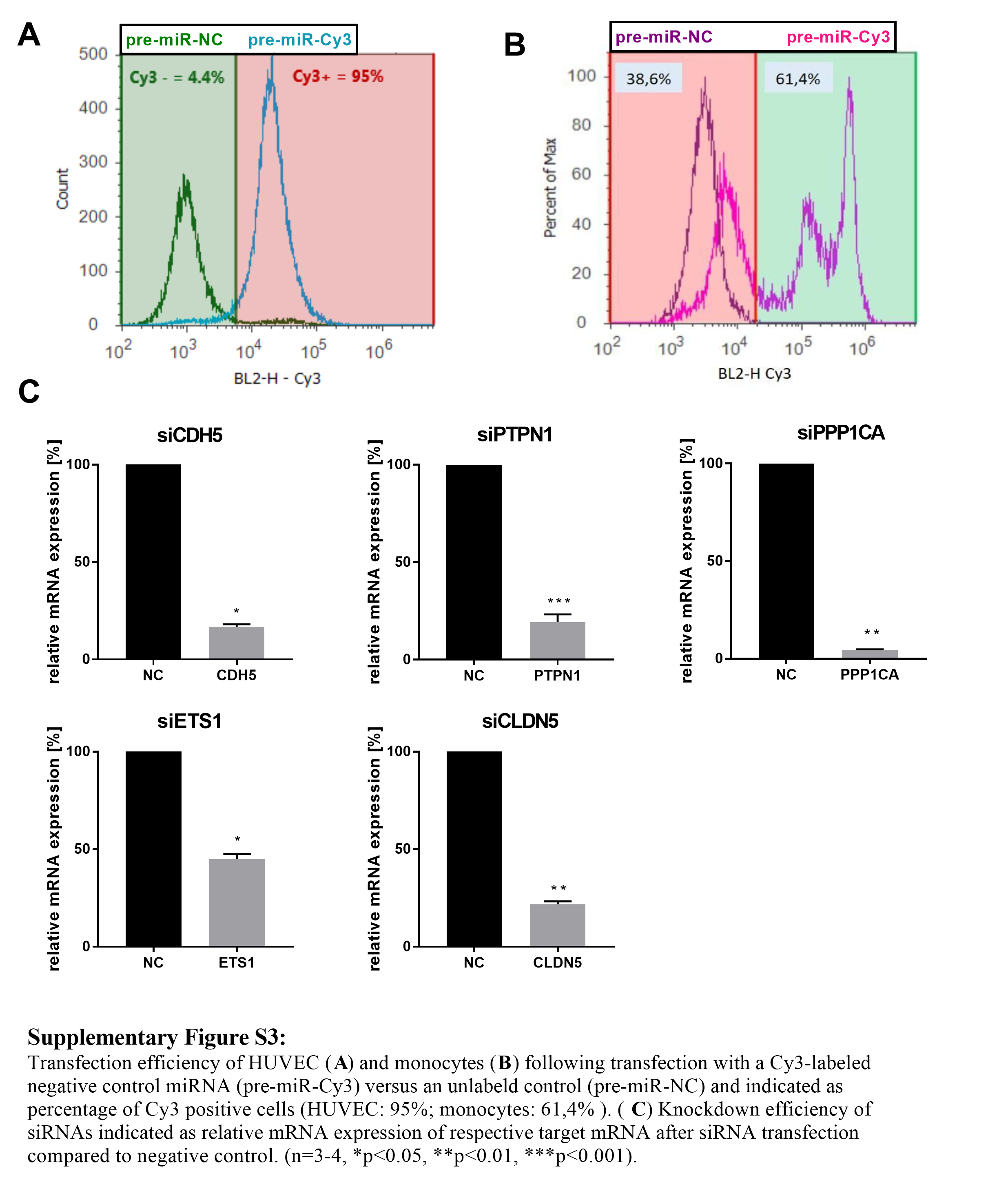

Supplement: Supplementary file 1 [file DataSheet_1.zip › Supplementary Material/Supplementary Figure S3.TIF]
